# Radiofrequency ablation (RFA) in unresectable pancreatic adenocarcinoma: meta-analysis & systematic review

**DOI:** 10.1007/s00464-024-11450-1

**Published:** 2024-12-10

**Authors:** Mathias Birrer, Baraa Saad, Susanne Drews, Charlotte Pradella, Mariana Flaifel, Emmanouil Charitakis, Niklas Ortlieb, Amanda Haberstroh, Vincent Ochs, Stephanie Taha-Mehlitz, Emanuel Burri, Andres Heigl, Daniel M. Frey, Philippe C. Cattin, Michael D. Honaker, Anas Taha, Robert Rosenberg

**Affiliations:** 1 Department of Visceral Surgery, Cantonal Hospital Baselland, Liestal, Switzerland; 2 Faculty of Medicine, University of Basel, Basel, Switzerland; 3 School of Medicine, St George’s University of London, London, UK; 4 Medoc Swiss, Science and Statistics, Basel, Switzerland; 5 Laupus Health Sciences Library, East Carolina University, Greenville, NC USA; 6 Department of Biomedical Engineering, Faculty of Medicine, University of Basel, Allschwil, Switzerland; 7 Clarunis, Department of Visceral Surgery, University Center for Gastrointestinal and Liver Diseases, St. Clara Hospital and University Hospital Basel, Basel, Switzerland; 8 Department of Gastroenterology and Hepatology, Medical University Clinic, Cantonal Hospital Baselland, Liestal, Switzerland; 9 Department of Surgery, Klinik-Impuls, Zurich, Switzerland; 10 Department of Surgery, Brody School of Medicine, East Carolina University, Greenville, NC USA

**Keywords:** Pancreatic adenocarcinoma, Radiofrequency ablation, Unresectable pancreatic adenocarcinoma, Survival analysis, Meta-analysis

## Abstract

**Background:**

Pancreatic adenocarcinoma remains a challenging malignancy with a poor prognosis. Radiofrequency ablation (RFA) has emerged as a potential treatment for unresectable pancreatic adenocarcinoma (UPAC) aimed at improving survival and quality of life. This meta-analysis and systematic review evaluates the outcomes of RFA in UPAC.

**Methods:**

A comprehensive search was conducted in MEDLINE, Embase, Scopus, and Cochrane Central databases from inception to October 2023. Studies included patients over 18 years with UAPC undergoing RFA. Survival rates and complication rates were assessed as primary outcomes. Data were pooled using random-effects models, and heterogeneity was assessed with *I*^2^ statistics. ROBINS-I tool was used for quality assessment.

**Results:**

Nine studies encompassing 265 patients met the inclusion criteria. The mean age was 64.5 years, with 42.5% female participants. Survival analysis showed that at 30 days post-RFA, the mortality rate was 3.3%. At 6 months, the mortality rate was 20.9%, increasing to 50.4% at 12 months. At 24 months, the mortality rate was 61.9%. The pooled mean survival period at 12 and 24 months was 9.18 months and 14.26 months, respectively. Overall, 78.4% of patients died during the follow-up period, with an overall mean survival period of 12.27 months. The most common were intra-abdominal (10.1%), pancreatic (9.8%), and hepatobiliary (6.7%) complications.

**Conclusions:**

RFA shows potential in the management of unresectable pancreatic adenocarcinoma, with a manageable safety profile. However, the high heterogeneity and risk of bias in available studies highlight the need for well-designed randomized controlled trials to confirm these findings and establish standardized protocols.

**Supplementary Information:**

The online version contains supplementary material available at 10.1007/s00464-024-11450-1.

Pancreatic cancer is the third leading cause of cancer-related death in the United States and remains a major cause of cancer deaths worldwide [1, 2]. Pancreatic cancer has several histological subtypes, with 90% being pancreatic ductal adenocarcinoma (PDAC) [1, 3]. The most widely clinically used system for classifying pancreatic cancer classifies tumors as resectable, borderline resectable, and locally advanced [4]. Unresectable pancreatic adenocarcinoma is defined as PDAC that invades adjacent structures, primarily vascular or digestive, and comprises unresectable locally advanced pancreatic tumors and unresectable metastatic tumors [5, 6].

Most PDAC patients present with advanced-stage cancer, with 80–85% being considered surgically unresectable at diagnosis [1, 7]. This results in a low 5-years survival rate of 12% [8], despite advancements in oncological treatment [1, 9]. The current management for patients with unresectable pancreatic adenocarcinoma (UPAC) mainly focuses on local tumor control, and on palliative measures to improve overall survival and quality of life [5]. Management for UPAC, and selected metastatic PDAC, may include systemic chemotherapy, radiotherapy, or a combination of chemoradiotherapy, with the possibility of neoadjuvant treatment and subsequent resection [10, 11]. Symptom control measures, like stenting or biliary-enteric bypass for blocked bile ducts, can also be implemented [7].

Radiofrequency ablation (RFA) is a minimally invasive procedure that uses high-frequency electric currents delivered through a needle electrode to heat and destroy nearby tumor tissue (coagulative necrosis) with minimal damage to surrounding areas [12]. The primary aim is local tumor control and symptom relief associated with tumor growth. RFA may also be combined with radio-chemotherapy, potentially improving outcomes. Although RFA has long been recognized as a potential therapy [13, 14], recent studies, such as those included in this review, show increasing interest in its use in unresectable pancreatic tumors. Several approaches have been established for the delivery of RFA: intraoperative, ultrasound-/imaging-guided percutaneous, ultrasound-guided endoscopic, or retrograde cholangiopancreatography endoscopic deliveries [15]. However, multiple factors limit the widespread use of RFA in pancreatic cancer compared to, for example, hepatic cancer [16]. These factors include the complex anatomy of the pancreas, which creates technical challenges [17], the close proximity of critical blood vessels and bile ducts that can be damaged by heat [18], and the increased risk of complications like pancreatitis due to the pancreas’ sensitive tissue [19]. All mentioned factors contribute to the fact that the evidence examining the safety of RFA and survival after the procedure of unresectable pancreatic cancer is not as robust [15] as, e.g., in hepatocellular- and cholangiocarcinoma, where guidelines and protocols [20] are well established. Due to a lack of robust evidence, clinicians are adopting this practice with caution.

A meta-analysis is urgently needed to assess RFA for unresectable pancreatic cancer. While interest is high, high-quality clinical studies are scarce. This analysis combines data from existing research to provide a clearer picture of RFA’s clinical safety and effectiveness. Notably, to our knowledge, no prior meta-analysis has examined RFA for unresectable pancreatic adenocarcinoma. Our study aims to fill this gap by evaluating RFA’s safety and impact on survival, for both primary and combination therapy of unresectable pancreatic adenocarcinoma.

## Methods

### Search strategy and data sources

A comprehensive search of MEDLINE (PubMed), Embase (Elsevier), Scopus (Elsevier), and Cochrane Central (Ovid) databases from inception to October 2023 was conducted. The search strategy, designed and conducted by a medical reference librarian (AH), involved keywords and subject headings for concepts including “radiofrequency ablation,” “pancreatic cancer,” “pancreatic neoplasms,” “pancreatic ductal adenocarcinoma,” and “pancreatic adenocarcinoma.” The review was registered prospectively with PROSPERO (CRD42023475406). Database results were uploaded into Covidence review software where deduplication took place. In accordance with Cochrane systematic review guidelines, two reviewers (MF and EC) screened titles, abstracts, and full texts based on the predefined eligibility criteria below. Conflicts were resolved by an independent third reviewer (BS).

### Eligibility criteria and quality assessment

Eligible studies must have met all the following inclusion criteria: (1) participants older than 18 years with unresectable pancreatic adenocarcinoma; (2) participants undergoing Radiofrequency ablation (RFA) as an intervention; (3) studies reporting primary outcomes of tumor response to RFA, short-term, and long-term survival and mortality rates, or complications/adverse events. Pancreatic adenocarcinoma was histologically confirmed in all included studies. Unresectable tumors were radiologically confirmed tumors invading adjacent structures, primarily vascular or digestive. In cases where RFA was the primary intervention, unresectable disease was determined at time of diagnosis. In cases where RFA was the secondary intervention, unresectable disease was determined following primary therapy. Randomized control trials, prospective, and retrospective cohort studies were included. Case reports, abstracts, and poster presentations were excluded. The methodological quality of each study was independently evaluated by two authors using the ROBINS-I [21] tool for non-randomized studies and RoB 2 [22] tool for randomized studies.

### Statistical analysis

For single-arm analyses, means of continuous variables and rates of binary variables were pooled using the random-effects model, generic inverse variance method of Der Simonian, Laird [23]. Proportions underwent logit transformation prior to meta-analyses. For two-arm analyses, pooled means and proportions were analyzed using an inverse variance method for continuous data and the Mantel–Haenszel method for dichotomous data. The weight of each study was assigned based on its variance. The heterogeneity of effect size estimates across the studies was quantified using the Q statistic and the *I*^2^ index. An *I*^2^ value of 0–25% indicates minimal heterogeneity, 26–50% moderate heterogeneity, and 51–100% substantial heterogeneity. Data analysis was performed using Open Meta analyst software (CEBM, Brown University, Providence, Rhode Island, USA). If mean or standard deviation (SD) was unavailable, the median was converted to mean and the range, interquartile range, or confidence intervals were converted to SD using the formulas from the Cochrane Handbook for Systematic Reviews of Interventions [24]. When possible, survival data were generated and reproduced using an algorithm that closely approximates the original individual patient time-to-event data from published Kaplan Meier survival curves [25].

### Endpoints

Tumors involving the head were defined as any tumor with head of the pancreas involvement, either alone or with other parts. This category included head only, head-uncinate, and head-body involvement. Similarly, the category of tumors involving the tail included tail only and body-tail involvement. Tumors not involving the head or tail included body only, uncinate only, neck only, uncinate-body, and neck-body involvement. Intraabdominal and gastrointestinal complications were defined as abdominal pain, seroperitoneum, abdominal infections, duodenal injury, duodenal bleeding, duodenal stricture, cystic fluid collection, or intra-abdominal abscess. Pancreatic complications were defined as increased amylase, acute pancreatitis, pancreatic fistulas, and peripancreatic effusion. Hepatobiliary complications were defined as portal vein thromboses, biliary leak, jaundice, biliary fistula, ascites, and hepatic insufficiency. Systemic and infectious complications were defined as pain, fever, vomiting, diarrhea, pneumonia, sepsis, wound infection, psychosis, and postoperative delirium. Survival period was defined as the period between date of RFA application and the date of longest known survival of patients. The mortality rate was defined as number of patients who died after undergoing RFA during a specific period divided by the total number of patients who were known to have completed follow-up in said period.

## Results

### Study selection

The initial search yielded 1623 potentially relevant articles from which 9 unique studies involving 265 patients met the eligibility criteria [19, 26–33]. The details of the study selection process and PRISMA (Preferred Reporting Items for Systematic Reviews and Meta-Analyses) flow diagram are depicted in Supplementary Fig. 1.

### Risk of bias

Results of the quality assessment of all included studies are shown in Supplementary Fig. 2. 6 studies were judged to have a moderate risk of bias [26, 27, 29–32], while 3 studies were judged to have a serious risk of bias [19, 28, 33].

### Baseline and procedural characteristics

The baseline characteristics of the included studies are comprehensively described in Table 1. 265 patients underwent 293 RFA ablations. 107 out of 252 (42.46%) patients were female [19, 26–33]. The mean age of participants was 64.5 years (95% CI 63.21, 65.77; *I*^2^ = 19.98%) [19, 26–28, 30–33]. 120 out of 190 tumors involved the head of the pancreas (60.5%; 95% CI 0.465, 0.746; *I*^2^ = 71.29%), while 49 out of 190 tumors involved the tail (18.3%; 95% CI 0.066, 0.300; I^2^ = 79.25%), and 21 out of 190 did not involve the head or tail (18.2%; 95% CI 0.062, 0.302; *I*^2^ = 83.37%) [19, 26, 27, 29, 31–33]. Out of 84 patients, 13 had stage II disease (13.7%; 95% CI 0.044, 0.354; *I*^2^ = 61.48%), 59 had stage III disease (72.3%; 95% CI 0.524, 0.923; *I*^2^ = 82.43%), and 12 had stage IV disease (11.6%; 95% CI 0.016, 0.217; *I*^2^ = 66.8%) [26, 27, 29, 30, 32, 33]. All tumors in this study were reported as unresectable (265/265, 100%) [19, 26–33]. RFA was delivered intraoperatively in 5 studies [27–30, 32], and via Endoscopic Ultrasound (EUS) in 4 studies [19, 26, 31, 33]. Procedural characteristics, therapy details, and medications are described in Table 2. The pooled mean size of tumors (*n* = 168) was 38.81 mm (95% CI 35.45, 42.18; *I*^2^ = 48.47%) [19, 26, 27, 31–33], and the pooled mean surface area (*n* = 36) was 14.26 mm^2^ (95% CI 9.16, 19.37; *I*^2^ = 62.04%) [19, 26, 31]. Pooled mean RFA application time was 3.59 min (n = 51) (95% CI 1.41, 5.76; I^2^ = 96.45%) [26, 31–33]. Pooled mean hospital stay was 3.3 days (*n* = 49) (95% CI − 0.174, 6.765; *I*^2^ = 100%) [19, 26, 31–33]. Out of 202 patients, 52.3% received only neoadjuvant chemotherapy prior to RFA (95% CI: 0.131, 0.914; *I*^2^ = 98.89%), while 19.2% received neoadjuvant chemotherapy and radiotherapy (95% CI 0.087, 0.298; *I*^2^ = 84.83%) [19, 26, 27, 29–31, 33]. Out of 198 patients, 35.1% received only adjuvant chemotherapy after RFA (95% CI 0.00, 0.705; *I*^2^ = 98.6%), while 13.7% received adjuvant chemotherapy and radiotherapy (95% CI 0.00, 0.292; *I*^2^ = 94.99%) [19, 27, 29–33]. Out of 63 reported patients, all received antibiotics (63/63, 100%) [19, 26–33].

**Table 1:** Baseline characteristics of included studies and patients

Study	Country	Type of study	Number of patients	Number of RFAs	Mean Age ± SD	Gender female (%)	Stage of disease				Tumor location		
							II B (%)	III (%)	IV (%)	Unresectable (%)	Involving the Head (%)	Involving the tail (%)	Not involving the head or tail (%)
Arcidiacono 2012	Italy/Germany	Prospective	16	16	65.6 ± 9.6	7 (44%)	0 (0%)	16 (100%)	0 (0%)	16 (100%)	9 (56%)	1 (6%)	6 (38%)
Cantore 2012	Italy	Prospective	107	107	64.1 ± 9.0	47 (44%)	NR^a^	NR	NR	107 (100%)	74 (69%)	33 (31%)	0 (0%)
D’Onofrio 2016	Italy	Retrospective	51	51	64. ± 11.5	24 (47%)	NR	NR	NR	51 (100%)	NR	NR	NR
He 2020	China	Retrospective	22	22	NR	9 (41%)	9 (41%)	13 (59%)	0 (0%)	22 (100%)	10 (45%)	8 (37%)	4 (18%)
Hlavsa 2019	Czech Republic	Prospective	24	24	64.8 ± 8.5	8 (33%)	4 (17%)	15 (62%)	5 (21%)	24 (100%)	NR	NR	NR
Scopelliti 2018	Italy	Prospective	10	14	62 ± 7.0	3 (30%)	NR	NR	NR	10 (100%)	4 (40%)	0 (0%)	6 (60%)
Spiliotis 2007	Greece	Retrospective	12	24	68.3 ± 5.7	6 (50%)	0 (0%)	8 (67%)	4 (33%)	12 (100%)	11 (92%)	0 (0%)	1 (8%)
Testoni 2022	Italy	Retrospective	13	13	64.8 ± 7.8	NR	NR	NR	NR	13 (100%)	8 (62%)	5 (38%)	0 (0%)
Thosani 2022	USA	Prospective	10	22	62.3 ± 5.7	3 (30%)	0 (0%)	7 (70%)	3 (30%)	10 (100%)	4 (40%)	2 (20%)	4 (40%)

^a^*NR* Not Reported

**Table 2:** Procedural Characteristic, therapy details and medications

Study	Number of RFAs	Tumor Size		Mean RFA Application Time (Min) ± SD	Mean Hospital Stay (Days) ± SD	Neoadjuvant therapy		Adjuvant therapy		Other Medications	
		Mean Size (mm) ± SD	Mean Area (cm^2^) ± SD			Chemo (%)	Chemo + Radiotherapy (%)	Chemo (%)	Chemo + Radiotherapy (%)	Antibiotic prophylaxis (%)	Other^b^
Arcidiacono 2012	16	35.5 ± 7.5	11.3 ± 4.4	1.78 ± 1.43	5.18 ± 0.4	10 (62%)	6 (38%)	NR	NR	16 (100%)	16
Cantore 2012	107	37.2 ± 13.4	NR^a^	NR	NR	49 (82%)	11 (18%)	45 (42%)	62 (58%)	NR	NR
D’Onofrio 2016	51	NR	NR	NR	NR	NR	NR	NR	NR	NR	NR
He 2020	22	NR	NR	NR	NR	22 (100%)	0 (0%)	22 (100%)	0 (0%)	NR	NR
Hlavsa 2019	24	NR	NR	NR	NR	0 (0%)	0 (0%)	13 (100%)	0 (0%)	24 (100%)	72
Scopelliti 2018	14	49.2 ± 14.2	20.7 ± 13.2	4.51 ± 2.39	3 ± 1.16	5 (50%)	5 (50%)	0 (0%)	0 (0%)	10 (100%)	20
Spiliotis 2007	24	45.4 ± 21.5	NR	6.08 ± 1.34	NR	NR	NR	0 (0%)	0 (0%)	NR	12
Testoni 2022	13	37.9 ± 13.0	NR	2.08 ± 1.25	5 ± 0	5 (38%)	8 (62%)	5 (38%)	3 (23%)	13 (100%)	2
Thosani 2022	22	39.2 ± 17.1	14.4 ± 10.6	NR	0 ± 0	7 (70%)	3 (30%)	0 (0%)	0 (0%)	NR	NR

^a^*NR* Not Reported

^b^Medications included—Gabexate mesilate, Indomethacin, Octreotide, Omeprazole, DVT prophylaxis, Somatostatin

### Outcomes of RFA ablation

**Fig. 1:**
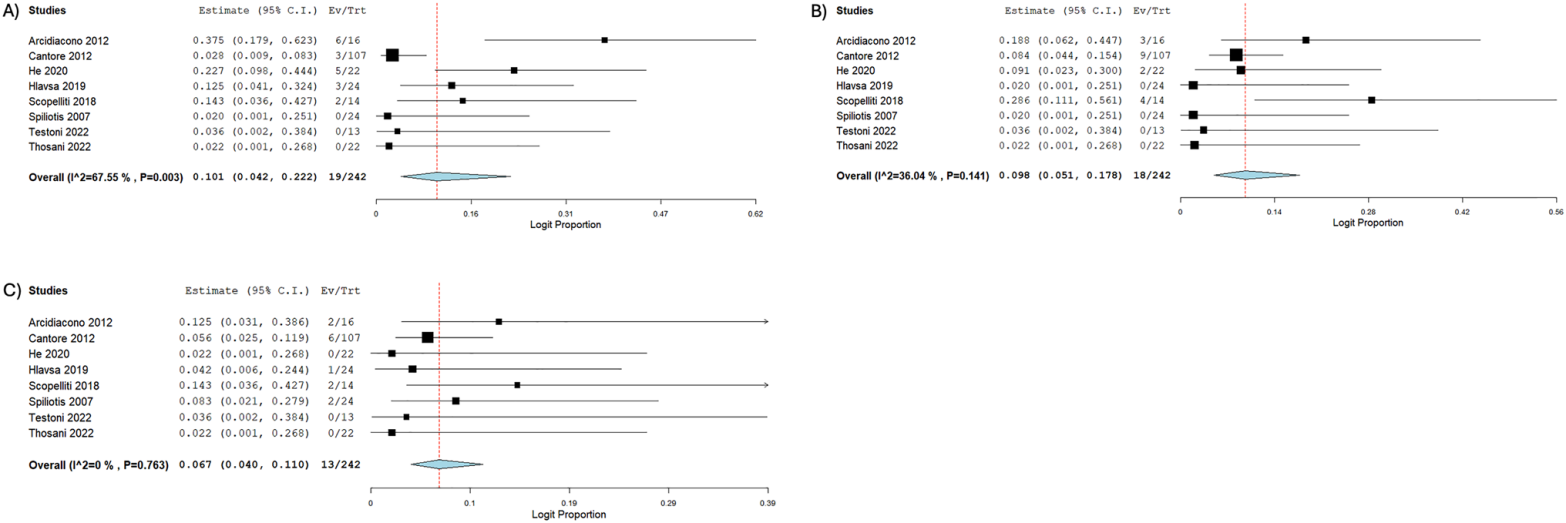
Forest plots of complications. **A** Intraabdominal and Gastrointestinal complications, **B** Pancreatic complications, **C** Hepatobiliary complications

Tumor marker measurements and complications of RFA are outlined in Table 3. In 242 procedures, 106 complications occurred (43.8%); of which 19 were intra-abdominal and gastrointestinal complications (10.1%; 95% CI 0.042, 0.222; *I*^2^ = 67.55%), 18 were pancreatic complications (9.8%; 95% CI 0.051, 0.178; *I*^2^ = 36.04%), and 13 were hepatobiliary complications (6.7%; 95% CI 0.040, 0.110; *I*^2^ = 0%) [19, 26, 27, 29–33]. 43 systemic or infectious complications were reported with 18 incidents of abdominal pain, 11 incidents of fever, and 3 incidents of sepsis. Forest plots of complications are depicted in Fig. 1. The pooled mean CA19-9 pre-RFA was 460.1U/ml (95% CI 160.85, 759.25; *I*^2^ = 73.83%) [19, 27, 28, 31, 33]. The pooled mean CA19-9, 30-day post-RFA, was 600.1U/ml (95% CI 357.13, 843.03; *I*^2^ = 0%) [28, 31]. There was no difference observed between pre-RFA and post-RFA CA19-9 levels (− 123.65 U/ml, 95% CI − 431.28, 183.97) [28, 31].

**Table 3:** Tumor markers measurements and complications of RFA

Study	No. of Patients	No. of RFA	Tumor markers		RFA related complications				Sum Of Complications Related To Other Procedures (%)	Total Number of Complications
			Mean Pre-RFA CA19-9 (U/ml) ± SD	Mean Post-RFA CA19-9 (U/ml) ± SD	Systemic & infectious	Intraabdominal & GI	Hepatobiliary system	Pancreatic		
Arcidiacono 2012	16	16	NR^a^	NR	0	6	2	3	1	12
Cantore 2012	107	107	147.8 ± 752.6	NR	1	3	6	9	9	30
D’Onofrio 2016	51	51	611.3 ± 954.4	582.6 ± 894.9	NR	NR	NR	NR	NR	NR
He 2020	22	22	NR	NR	36	5	0	2	NR	43
Hlavsa 2019	24	24	NR	NR	5	3	1	0	1	10
Scopelliti 2018	10	14	1718.9 ± 3161.1	1393.4 ± 2670.6	0	2	2	4	NR	8
Spiliotis 2007	12	24	NR	NR	1	0	2	0	NR	3
Testoni 2022	13	13	354 ± 521.9	NR	0	0	0	0	NR	0
Thosani 2022	10	22	923.2 ± 1078.6	1327.2 ± NR	0	0	0	0	NR	0

^a^*NR* Not Reported

**Fig. 2:**
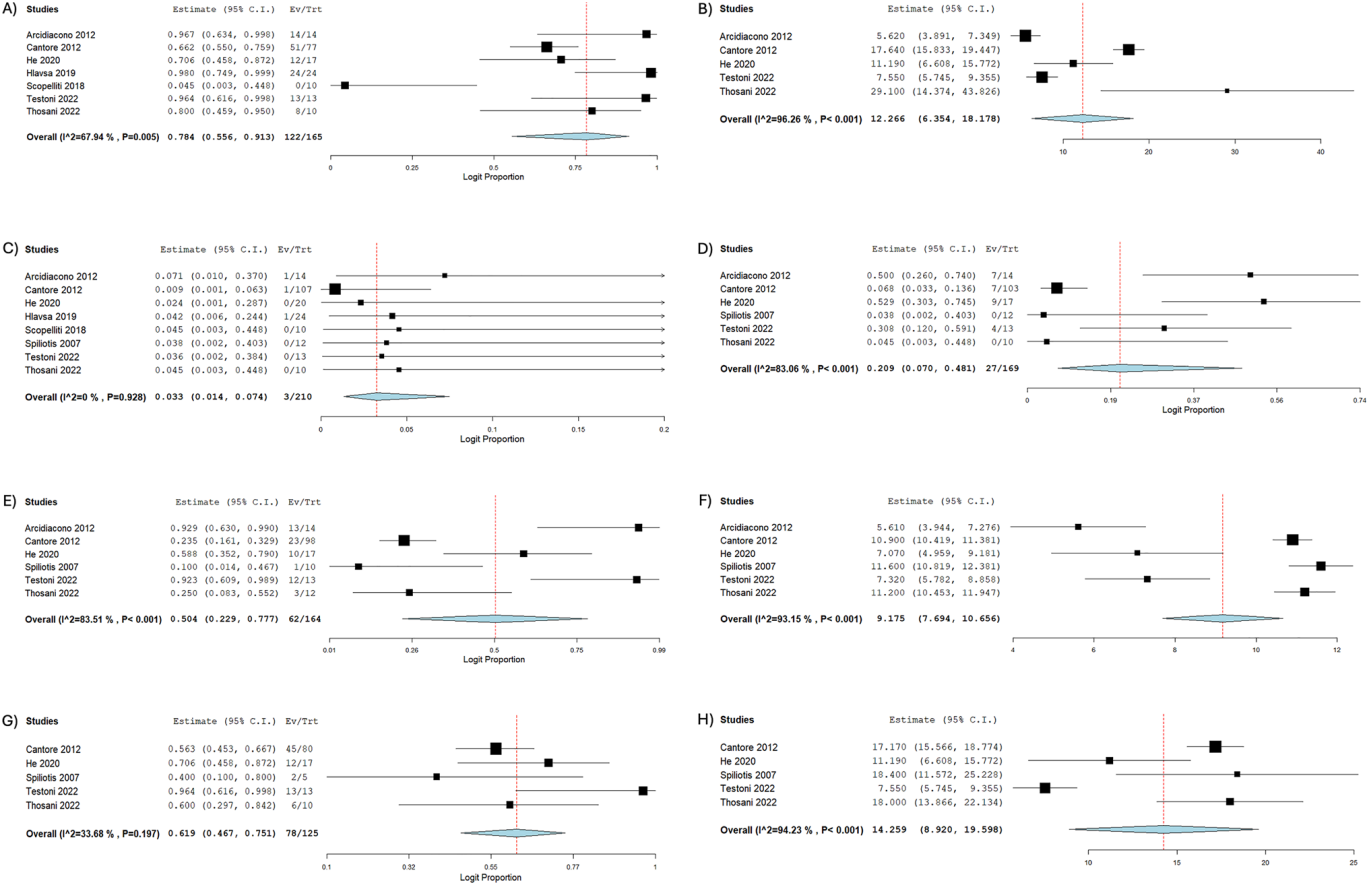
Forest plots of survival, **A** Overall Mortality, **B** Overall Mean survival period (in months), **C** 30-day mortality, **D** 6-month mortality, **E** 12-month mortality, **F** Mean survival period (at 12 months, in months), **G** 24-month mortality, **H** Mean survival period (at 24 months, in months)

The survival data post-RFA are presented in Table 4. Out of the initial 265 enrolled patients, 165 were included in this meta-analysis for the overall survival assessment after accounting for those lost to follow-up. The average longest follow-up per study was 25.9 ± 24 months [19, 26–33]. Within the first 30 days post-RFA, 3 deaths were recorded (*n* = 210) (3.3%; 95% CI 0.01, 0.07, *I*^2^ = 0%), while 207 patients survived this period (*n* = 210) (96.7%; 95% CI 0.93, 0.99; *I*^2^ = 0%) [19, 26, 27, 29–33]. At 6 months, 27 deaths were recorded (*n* = 169) (20.9%; 95% CI: 0.07, 0.48; I^2^ = 83.06%), whereas 142 survived (*n* = 169) (79.1%; 95% CI: 0.52, 0.93; I^2^ = 83.06%) [19, 26, 27, 29, 32, 33]. At 12 months, 62 deaths were recorded (n = 164) (50.4%; 95% CI: 0.23, 0.78; I^2^ = 83.51%), whereas 102 patients were observed to be alive (*n* = 164) (49.6%; 95% CI 0.22, 0.77; *I*^2^ = 83.51%) [19, 26, 27, 29, 32, 33]. The pooled mean survival period at 12 months was 9.18 months (95% CI 7.69, 10.66; *I*^2^ = 93.15%) [19, 26, 27, 29, 32, 33]. At 24 months, 78 deaths were recorded (*n* = 125) (61.9%; 95% CI 0.47, 0.75; *I*^2^ = 33.68%), whereas 47 patients were observed to be alive (*n* = 125) (38.1%; 95% CI 0.25, 0.53; *I*^2^ = 33.68%) [19, 27, 29, 32, 33]. The pooled mean survival period at 24 months was 14.26 months (95% CI 8.92, 19.6; *I*^2^ = 94.23%) [19, 27, 29, 32, 33]. Overall, out of 165 patients, 122 deaths were recorded (78.4%; 95% CI 0.56, 0.91; *I*^2^ = 67.94%) and 43 patients were alive at the end of the follow-up period (21.6%; 95% CI 0.09, 0.44; *I*^2^ = 67.94%) [19, 26, 27, 29–31, 33]. The overall mean period of survival observed was 12.27 months (95% CI: 6.35, 18.18, *I*^2^ = 96.26%) [19, 26, 27, 29, 33]. Forest plots of survival data are depicted in Fig. 2.

**Table 4:** Survival data post-RFA

Study	Longest follow-up time	Overall Survival			30-Day mortality		6-Months survival		12-Months survival			24-Months survival		
		Mean Survival (Months) ± SD	Deaths (%)	Patients alive (%)	Deaths (%)	Patients alive (%)	Deaths (%)	Patients alive (%)	Mean survival (Months) ± SD	Deaths (%)	Patients alive (%)	Mean survival (Months) ± SD	Deaths (%)	Patients alive (%)
Arcidiacono 2012	12 months	5.62 ± 3.18	14 (100%)	0 (0%)	1 (7%)	13 (93%)	7 (50%)	7 (50%)	5.61 ± 3.18	13 (93%)	1 (7%)	NR	NR	NR
Cantore 2012	25 months	17.64 ± 8.09	51 (66%)	26 (34%)	1 (1%)	106 (99%)	7 (7%)	96 (93%)	10.9 ± 2.43	23 (23%)	75 (77%)	17.17 ± 7.32	45 (56%)	35 (44%)
D’Onofrio 2016	1 month	NR	NR	NR	NR	NR	NR	NR	NR	NR	NR	NR	NR	NR
He 2020	25 months	11.19 ± 9.64	12 (71%)	5 (29%)	0 (0%)	20 (100%)	9 (53%)	8 (47%)	7.07 ± 4.44	10 (59%)	7 (41%)	11.19 ± 9.64	12 (71%)	5 (29%)
Hlavsa 2019	25 months	9.9 ± NR	24 (100%)	0 (0%)	1 (4%)	23 (96%)	NR	NR	NR	NR	NR	NR	NR	NR
Scopelliti 2018	1 month	NR	0 (0%)	10 (100%)	0 (0%)	10 (100%)	NR	NR	NR	NR	NR	NR	NR	NR
Spiliotis 2007	38 months	NR	NR	NR	0 (0%)	12 (100%)	0 (0%)	12 (100%)	11.6 ± 1.26	1 (10%)	9 (90%)	18.4 ± 7.79	2 (40%)	3 (60%)
Testoni 2022	25 months	7.55 ± 3.32	13 (100%)	0 (0%)	0 (0%)	13 (100%)	4 (31%)	9 (69%)	7.32 ± 2.83	12 (92%)	1 (8%)	7.55 ± 3.32	13 (100%)	0 (0%)
Thosani 2022	81 months	29.1 ± 23.76	8 (80%)	2 (20%)	0 (0%)	10 (100%)	0 (0%)	10 (100%)	11.2 ± 1.32	3 (25%)	9 (75%)	18 ± 6.67	6 (60%)	4 (40%)

^a^*NR* Not Reported

## Discussion

The frequent presentation of PDAC as an unresectable disease limits surgical options [34]. To address these limitations with surgery, various methods to destroy tumors locally (ablation) have been developed. These include stereotactic body radiation therapy, irreversible electroporation, cryotherapy, microwave therapy, and high-intensity focused ultrasound [35].

Radiofrequency ablation (RFA) shows considerable promise as a potential treatment for non-resectable pancreatic cancer. Its safety and effectiveness have already been established in managing hepatocellular carcinoma, esophageal dysplasia, and pancreatic neuroendocrine tumors [36–40]. Studies using contrast-enhanced EUS show RFA can enhance blood flow, improving chemotherapy effectiveness [41]. Additionally, RFA goes beyond cell death by potentially altering the tumor environment (neoplastic microenvironment) [42]. Studies show increased immune cell activity after RFA [43], suggesting an anti-tumor immune response. More research is needed to substantiate this mechanism [44], but RFA’s impact on the immune system warrants further exploration.

Some major concerns for using RFA for treating pancreatic cancer are as follows: (1) Anatomical limitations: The pancreas is located near vital organs like blood vessels and bile ducts. Precise placement of the RFA probe is crucial to avoid damaging these structures. The dense network of surrounding organs limits the safe zone for heat ablation, especially for larger tumors [15]. (2) Tumor characteristics: RFA is more effective on smaller, well-defined tumors. Pancreatic cancer is often diagnosed at later stages when tumors are larger and more diffuse (size of 3 cm for head, and 6 cm for body/tail at diagnosis) [45], making complete ablation difficult. (3) Complications: Potential complications of RFA in pancreatic cancer include bleeding, infection, pancreatitis (inflammation of the pancreas), and leakage of bile [15, 46].

This study presents the first-ever comprehensive meta-analysis evaluating RFA for treating UPAC. Our analysis offers the most extensive quantitative data to date on RFA’s safety and survival in this context. However, limited data with high heterogeneity characterizes this meta-analysis, yet it suggests promise for RFA in unresectable pancreatic cancer.

In our analysis, 30-day mortality rates appear low, and while pooled survival does not drastically improve over other treatments, a potential benefit is hinted at. When comparing our cohort to a cohort with local and resectable pancreatic cancer receiving R0 or R1 resection with adjuvant chemotherapy, studies show that surgery offers better survival (47.5% at 2 years, with a median survival length of 22.1 months) [47] compared to our pooled RFA statistics (61.9% mortality at 2 years and median survival at 14.26 months). It is important to note that this cannot be a direct comparison due to the differences in the stages of disease between the cohorts yet can rather provide contextual insight into the outcomes of RFA. In patients with locally advanced pancreatic adenocarcinoma receiving chemotherapy alone, the overall survival was 11.4 months [48], similar to the overall survival of the current study (12.3 months). The lack of a large observable difference may not justify the use of RFA as a standalone treatment. Multicenter randomized control trials are needed to directly compare RFA to standard protocols and to explore whether RFA significantly increases surgical candidacy. Additionally, standard protocols regarding adjuvant therapy, neoadjuvant therapy, as well as triple therapy approaches with RFA must be explored in greater detail to optimize the benefit of RFA in this population. The potential of RFA to downstage unresectable pancreatic cancer and increase surgical candidacy has been a recent subject of interest. A study that investigated downstaging tumors with the intent of radical resection in patients receiving multimodal chemoradiotherapy in combination with RFA showed that 7% of patients achieved radiological downstaging with a mean interval of 9 months from ablation to surgery [49]. Of these, 50% of patients were resected with radical intent, achieving R0 resections, while 50% of patients were judged as technically unresectable for severe peritumoral fibrotic reaction due to previous RFA and radiotherapy [49]. It was concluded that RFA did not significantly impact downstaging rates overall, but when downstaging is achieved, it appeared to provide a substantial survival benefit in resected patients [49]. Only one study in our meta-analysis reported downstaging of disease, with 2 out of 10 patients becoming eligible for potentially curative pancreaticoduodenectomy following RFA [19]. This highlights that while downstaging can offer a potential survival benefit, its occurrence and subsequent surgical success remain limited and further data are required to fully assess the utility of RFA in downstaging disease and increasing surgical candidacy.

Our pooled data reveal that in 242 procedures, 106 complications occurred, which may seem relatively high, and the occurrence of gastrointestinal, biliary, local pancreatic, and systemic complications reflects the discussed limitations of using RFA in the pancreas’ anatomy and location. It is important to note that different studies had different definitions of complications with some papers reporting only serious or RFA-specific complications, while others included pain, vomiting, diarrhea, and fever with no evidence of sepsis or intra-abdominal infection as complications. Examining the RFA complication rate in more detail reveals that for RFA interventions, 10.1% intra-abdominal and gastrointestinal, 6.7% hepatobiliary, and 9.8% pancreatic complications occurred. Previous well-established safe procedures using RFA in hepatocellular carcinoma, esophageal dysplasia, and pancreatic neuroendocrine tumors [36–40] showed comparable complication rates to those of our study, implying that treatment in UPAC can be considered relatively safe. What is more, the pooled 30-day mortality was 3.3%, indicating that RFA is not likely to directly cause mortality. Our study links RFA complications to heat damage on nearby organs. According to D’Onofrio et al., while artery infiltration often indicates a challenging resection, it does not contraindicate radiofrequency ablation (RFA) [28]. Even with tumor infiltration into mesenteric vessels or the duodenal wall, RFA can still be performed effectively [28]. The critical factor is ensuring that the necrotic area does not extend beyond the lesion, respecting safety margins around major vascular and digestive structures [28]. Additionally, the optimal settings (90 °C for 5 min) [50, 51] improved risk. Although some residual tumor tissue may remain, the procedure is feasible due to the ability to target the tumor in a way that avoids direct damage to major vessels. The presence of arterial flow around the tumor also helps protect vessels from excessive heat damage, making RFA a viable option even in cases with vascular involvement [28]. Additionally, RFA can stimulate systemic anti-tumor immune responses and create a proimmune environment, which is why it is reasonable to complete cytoreductive treatment by offering patients chemotherapy and/or radiotherapy [28]. On the other hand, although RFA is not directly linked to mortality, research is required to obtain data on long-term pain control and quality-of-life assessment, as the lack of data represents a drawback for the clinical utilization of RFA.

When examining the Ca19-9 data in our series, the pooled results showed no statistical difference preoperatively and postoperatively despite an observable decrease. In one study, an increase in Ca19-9 in 17/51 patients was reported [28]. This can be possibly explained by early measurements recording the cytolytic effect of the procedure or later measurements recording undetected systemic spread of the disease [28]. However, it is difficult to obtain a conclusion, as our data are limited due to the small sample size. A standardized protocol and clear serial measurements are warranted to explain the stability in Ca19-9 seen in our results.

RFA is not the only ablative technique that shows promise in pancreatic tumors. Irreversible electroporation (IRE) is a non-thermal ablation therapy that utilizes a high-voltage, low-energy electrical pulse to cause damage and cell death while preserving surrounding vital structures and targeting areas near sensitive organs [52]. IRE has several advantages compared to other ablative therapies including RFA. IRE spares blood vessels and bile ducts while avoiding the "heat sink" effect that can limit the efficacy of thermal-based treatments like RFA [29]. IRE has shown promising results in improving progression-free survival (PFS) and overall survival (OS) rates, especially when used after induction chemotherapy or in combination with RFA [29, 53]. On the other hand, IRE has several disadvantages including the challenge of precise placement of the electrode in complex tumor geometries [53, 54]. IRE is also associated with several complications including procedure-related pain, severe pancreatitis, cholangitis, and portal vein thrombosis [53, 54]. Despite these risks, IRE’s safety profile, with lower complication rates compared to RFA, makes it a feasible and increasingly preferred option in certain clinical scenarios.

The present study has several limitations. A potential limitation of our meta-analysis is the selection bias inherent to studies investigating RFA for unresectable pancreatic cancer. While our analysis suggests no greater selection bias compared to typical randomized controlled trials (RCTs), the possibility cannot be entirely eliminated. Notably, the selection criteria for choosing UPAC patients who receive RFA versus palliative radio/chemotherapy remain unclear. These two patient groups might differ in factors like overall health, tumor characteristics, or the presence of comorbidities. Such differences could influence survival outcomes, potentially impacting the conclusions drawn about RFA’s potential survival benefit. The heterogeneity in patient selection criteria across the included studies was notable introducing potential selection bias. While most studies selected patients based on the resectability of the tumor rather than the disease stage, there were variations in criteria, such as the exclusion of patients with distant metastases in some studies but not in others. On the other hand, our study’s participant demographics, with an average age of 64.5 years and a majority (60.5%) having tumors in the pancreatic head, align well with established characteristics of PDAC patients [7]. Additionally, the observed percentage of female patients (42.46%) falls within expected ranges [7]. These findings suggest our sample population could be a fair representation of the broader PDAC population. The heterogeneity in patient selection criteria across the included studies was notable.

Our findings may be limited by the significant variation in RFA application across the analyzed studies. Treatment regimens differed greatly, with various combinations of chemotherapy, radiotherapy, and surgical approaches employed. Additionally, some studies incorporated biliary bypass procedures, while others used endoscopic ultrasound (EUS-RFA) or laparoscopic RFA techniques. Standardized treatment protocols applied in large, randomized studies must be completed in order to understand the clinical and practical applications of RFA in UPAC. Furthermore, variations in probe types across studies could have impacted treatment duration, efficacy, and complication rates. Additionally, the lack a standardized grading scale for complications can lead to inconsistent reporting across studies making it difficult to compare and synthesize data and potentially introducing bias. A further limitation is the presence of loss to follow-up and censored data in the survival analysis. It is possible that this skews the overall survival estimate to a more pessimistic outcome, underestimating the true overall survival rate. Finally, the relatively new application of RFA in pancreatic cancer introduces potential bias as the learning curve for both endoscopists and surgeons was not evaluated. These factors contribute to the heterogeneity of our findings and highlight the need for further standardized approaches to RFA treatment for unresectable pancreatic adenocarcinoma.

## Conclusion

This meta-analysis suggests promise for RFA as a treatment option for unresectable pancreatic adenocarcinoma. While minimal trends toward improved survival compared to other treatments were observed, the generalizability of these findings is limited by the heterogeneity of included studies. Variations in patient selection, treatment regimens, and RFA techniques necessitate further investigation. Future research should focus on developing standardized protocols for RFA application specifically in UPAC. Prospective studies are needed to definitively assess the impact of RFA on survival in this population, considering factors like learning curve, probe type, and the potential benefits of combining RFA with other treatment modalities. By addressing these limitations, future research can clarify the role of RFA in optimizing treatment strategies for UPAC patients and determine its potential application for unresectable disease.

## Supplementary Information

Below is the link to the electronic supplementary material.

Supplementary file1 (PNG 263 KB)

Supplementary file2 (PNG 438 KB)

## Data Availability

With the publication, the data set used for this meta-analysis will be shared upon request from the study authors.
