# Supplementary figures and images for: Radiofrequency ablation (RFA) in unresectable pancreatic adenocarcinoma: meta-analysis & systematic review
Source: Surg Endosc. 2024 Dec 10;39(1):141–52. doi: 10.1007/s00464-024-11450-1 (PMC11666652; doi:10.1007/s00464-024-11450-1)

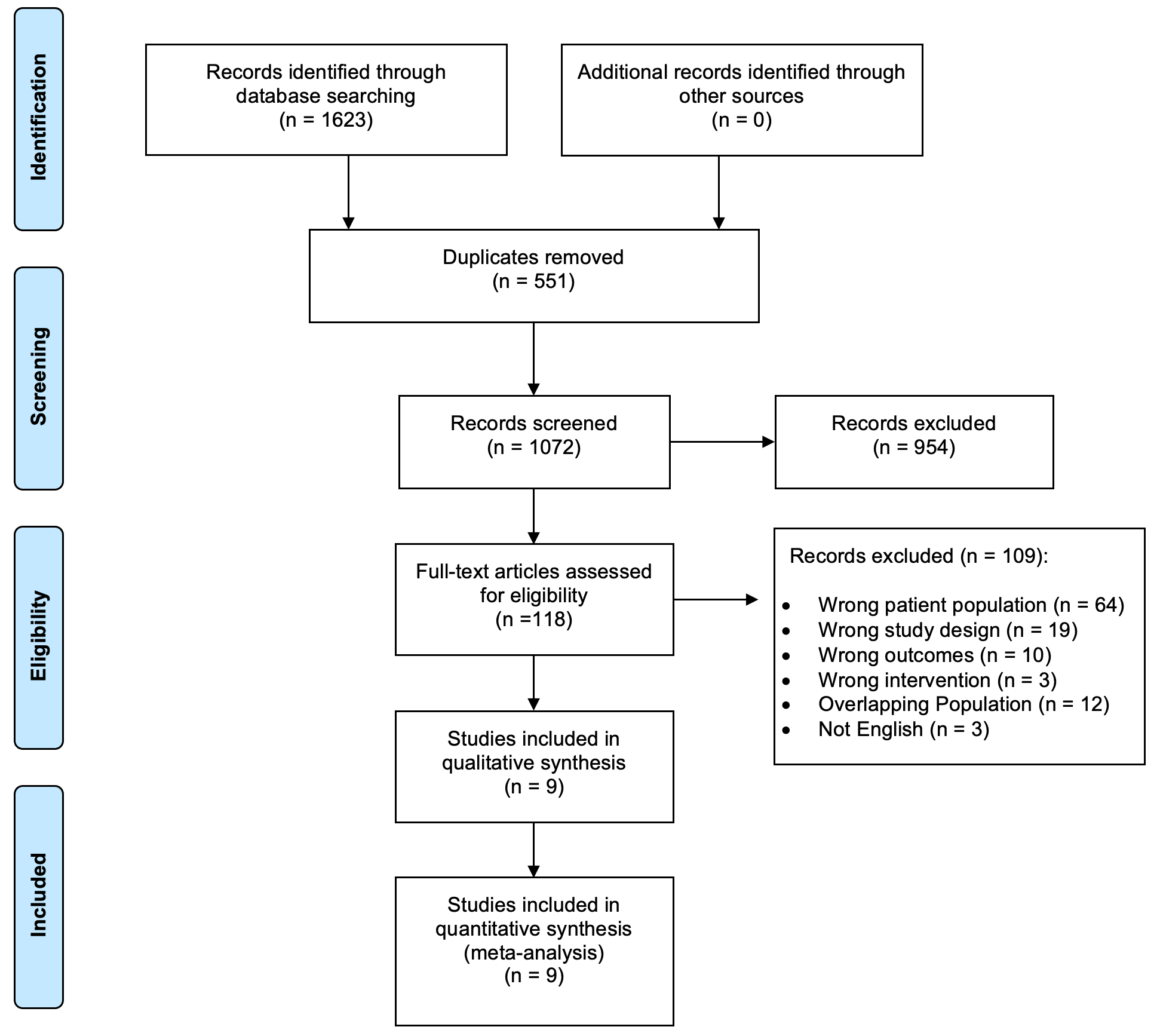

Supplement: Supplementary file 1 — Supplementary file1 (PNG 263 KB) [file 464_2024_11450_MOESM1_ESM.png]

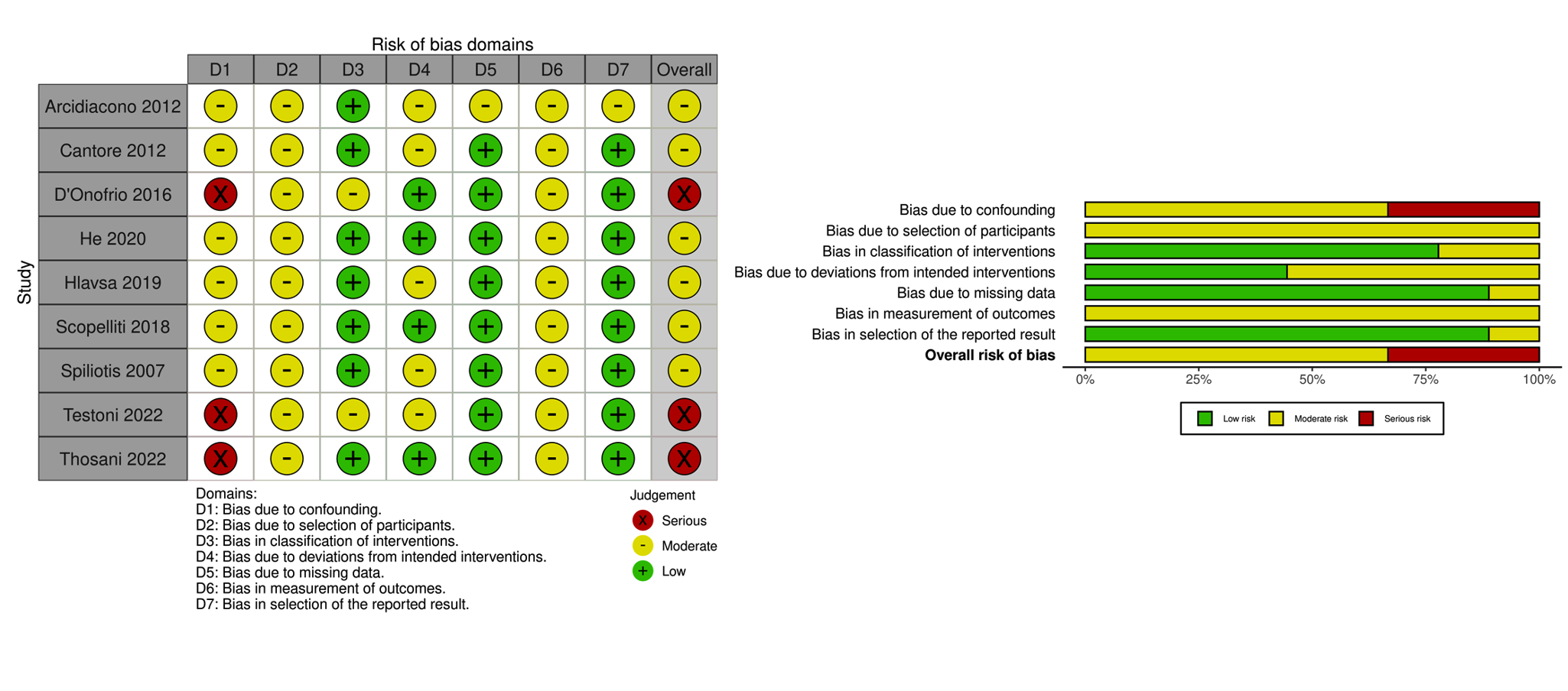

Supplement: Supplementary file 2 — Supplementary file2 (PNG 438 KB) [file 464_2024_11450_MOESM2_ESM.png]
